# The Centennial

**Published:** 1876-09

**Authors:** 


*The Centennial.*—Visits comprising some twelve days at three different periods, enabled us to see something of this truly *grand exposition* of the world's products and curiosities; one which has never been equalled, and which should be seen by every man, woman and child who can appreciate such a display in the civilized world.

The exhibition of dental articles in the United States Department is very creditable to all the exhibitors, and far surpasses anything of the kind from other countries.

Every dental instrument and appliance which enlightenment in the science has produced is here shown in the highest perfection.

Gold in all forms and qualities, some of which are astonishing as well as beautiful, is presented in the most attractive manner; and private cases of specimens of mechanical work are to be met with, some of which plainly indicate the great progress of the art in this branch of dental surgery.

Among the number is a case exhibited by Dr. John Allen, of New York City, which, in addition to many beautiful specimens of continuous gum work, some on platina plate, and others transferred to gold plate, contains valuable relics, such as are rarely met with outside of the museums or private collections to which they belong. Among these specimens are full upper and lower sets of artificial teeth worn by Gen. Washington, one of which, a double set, is from the Museum of the Baltimore College of Dental Surgery, and consists of teeth carved from ivory, and mounted on gold plates, the upper and lower sets being connected by spiral springs. In the ivory forming the bases of the artificial teeth. are as well defined carious cavities as can be found in natural teeth thus affected. This is the set of teeth which was worn by Gen. Washington longer than any other he had made, and it has been repaired several times.

Another curiosity is a lower set once worn by Gen. Washington, and made by Dr. Jno. Greenwood, and now owned by Mr. Isaac J. Greenwood, of New York, also carved from ivory and mounted on gold plate. This set, however, does not by its condition show the use to which the Baltimore College set has been subjected.

Another relic in this case is the last natural tooth which was extracted from the mouth of Gen. Washington, by Dr. John

Greenwood, and now owned by Mr. Isaac J. Greenwood, of N. Y. It is attached to a gold watch chain, and was thus worn by Dr. Greenwood for many years. In the same case is also an upper set of porcelain teeth and a lower set of çarved ivory teeth, mounted on tin bases, and which were worn by Aaron Burr. They are now in the possession of Dr. Tennison, of New York.

The collection of Aztec skulls in the department of Peru, will also prove highly interesting to medical and dental visitors, as well as many other specimens from remote regions, and of other ages to be found in the different parts of the Centennial buildings.

